# Evidence of dynamic visual acuity impairment in asymptomatic mixed martial arts fighters

**DOI:** 10.2217/cnc-2016-0032

**Published:** 2017-07-07

**Authors:** Merrill R Landers, Robert Donatelli, Jennifer Nash, Randa Bascharon

**Affiliations:** 1 Department of Physical Therapy, University of Nevada, Las Vegas, 4505 Maryland Parkway, Box 453029, Las Vegas, NV 89154, USA; 2 Las Vegas Physical Therapy & Sports (website: modernathleticscience.com), Las Vegas, NV 89117, USA; 3 Physical Therapy, Cleveland Clinic Lou Ruvo Center for Brain Health, Las Vegas, NV 89106, USA; 4 Orthopedic & Sports Medicine Institute of Las Vegas, Las Vegas, NV 89117, USA

**Keywords:** combat sports, concussion, vestibulo-ocular reflex

## Abstract

**Aim:**

The purpose of this study was to determine the amount of visual acuity loss with head movement in actively training mixed martial arts (MMA) fighters.

**Methods:**

Vestibulo-ocular reflex function of 22 asymptomatic, male MMA fighters (age = 29.2 ± 5.1) was assessed by taking the difference between static visual acuity and the dynamic visual acuity test, in both yaw and pitch planes.

**Results:**

The mean static visual acuity testing logMAR was -0.173 (standard deviation [SD] = 0.114). Mean dynamic visual acuity test values decreased with head movement to 0.196 logMAR (SD = 0.103) in yaw; p < 0.001, and to 0.283 logMAR (SD = 0.133) in pitch; p < 0.001.

**Conclusion:**

MMA fighters had a decay, beyond normal ranges, in visual acuity during head movement. These decreases may suggest vestibulo-ocular reflex impairment and were unrelated to self-reported concussion history. These results should be cautiously interpreted since there was not a control group.

Mixed martial arts (MMA) is a full contact combat sport that exposes the athlete to repeated head impact with subsequent head injury rates similar to boxing [1,2]. A recent meta-analysis by Lystad reported that MMA fighting resulted in an overall injury incidence rate of 228.7 injuries per 1000 athlete exposures; and, of these injuries, using the four studies used in this calculation, the percentages attributed to concussions were 2.0, 3.8, 10.0 and 20.4% [3]. Other studies not included in the Lystad *et al*. study have shown concussion rates ranging from 15.4 per 1000 athlete-exposures [4] to 0.7–250.6 per 1000 athlete-exposures for various combat sports [5]. One study by Buse found that the proportion of MMA fights stopped because of head impact was higher than in other full combat sports [6]. Taken together, these MMA-associated concussion rates warrant further investigation about the long-term effects of participation in the sport.

To investigate the long-term effects of repeated head impacts and concussion, one could test the function of the vestibular system which has been shown to be impaired in a high proportion of youth following concussion [7–9]. In the acute phase of a concussive event, the vestibular system is one of the main systems that is assessed, most typically by testing balance and evaluating vestibular-related symptoms [8,10]. Another way of testing vestibular function is by testing visual acuity during head movements, which is maintained by the vestibular ocular reflex (VOR). The VOR stabilizes images on the retina with head movement and has three main components: the peripheral sensory apparatus (vestibular system), a central processing mechanism and the motor output (the eye muscles). Visual acuity normally degrades with head movement; however, if this degradation is beyond normal ranges it may suggest impairment in the interaction of the vestibular, visuomotor and visual systems [11]. Thus, while assessment of the vestibular system provides important information about the extent of an acute concussive event, it may also provide valuable information about the long-term effects of that injury.

Although, most research in this area is fairly recent, there are several lines of evidence that the VOR is an important system to monitor as it appears to be related to the postconcussion recovery. Gottshall *et al*. have been able to associate recovery of VOR dysfunction in those recovering from mild head injury suggesting that recovery of VOR function may be one indicator of postconcussion recovery [12]. Corwin *et al*. reported that postconcussive vestibular symptoms are associated with a protracted recovery [7]. In support of the notion that the vestibular system is an important system to monitor, Ellis *et al*. have proposed an evidence-based classification system that includes three different postconcussion disorders, one of which is a vestibulo-ocular postconcussion disorder [13]. It seems logical, therefore, that an investigation of the function of the VOR in those with MMA fighting experience may shed some light into the long-term effects of MMA-related concussive and subconcussive head impacts.

Because, it is impractical to test fighters before and after exposure to MMA, this cross-sectional study on experienced MMA fighters is warranted and may provide evidence of the long-term effect of repeated head trauma on vestibular function. Thus, the primary purpose of this study was to determine if actively training MMA fighters exhibits a loss in visual acuity beyond normal ranges with head movement. Because MMA fighting exposes fighters to repeated head impacts, we hypothesized that the fighters may show deficits in vestibulo-ocular function and these deficits would be greater in those with a reported history of concussion.

## Methods

### Participants

Twenty-two male MMA fighters (mean age = 29.2, standard deviation [SD] = 5.1; mean years of MMA experience = 9.2, SD = 5.8) who were actively training and asymptomatic (e.g., no current neck injuries/pain, concussion or head injury, inner ear or upper respiratory tract infection, nystagmus, diagnosable vestibular disorder) participated in this study. The mean number of previously diagnosed concussions reported was 0.64 (SD = 0.73) and 10 of the 22 fighters did not report a concussion history. Of those with a concussion history, 11 out of the 12 reported a history of between 1 and 3 concussions whereas one participant reported a history of 6+ concussions. Participants, consented under Physiotherapy Associates Institutional Review Board approval, were recruited from an MMA training gym in Las Vegas, Nevada. All participants were actively training for competition and several were high-level professional MMA fighters at the time of data collection (2010 and 2011). Participants completed a screening questionnaire about their MMA experience, current health status and concussion history. At the time of participation, few of the participants were currently receiving treatment for headache (n = 0), migraine (n = 0), epilepsy/seizures (n = 0), substance abuse (n = 0), psychiatric problems (n = 1), depression (n = 1), ear infection (n = 0) and whiplash (n = 0). Few participants reported minor problems of an ongoing nature: nausea (n = 0), balance problems (n = 3), concentrating (n = 1), remembering (n = 1), sensitivity to light (n = 0), blurred vision (n = 0), nervousness (n = 0), numbness (n = 2), sleeping problems (n = 2), fatigue (n = 2), irritability (n = 1) and neck stiffness (n = 8).

### Overall design

A cross-sectional design was utilized wherein the function of the VOR was tested using the dynamic visual acuity test (DVAT). The DVAT was assessed using the NeuroCom InVision system (Natus, OR, USA) and conducted according to manufacturer's recommended protocol. This system and protocol have been reported to have good reliability in athletic populations [14,15]. Static visual acuity testing (SVAT) and the perception timed test (PTT) were conducted first followed by the DVAT in the following two planes: pitch (vertical movement – neck flexion and extension) and yaw (horizontal movement – left and right head rotation). SVAT and DVAT values are derived from the Snellen fraction and converted to log of minimum angle of resolution (logMAR) values.

### Procedures

#### Static visual acuity testing

The SVAT measures visual acuity with the head stationary and establishes a baseline for comparing the decay in visual acuity with head movement. With all testing, the participant sat 10 feet (3.3 m) from a computer screen (bridge of nose to computer screen). During the SVAT, the optotype (the letter ‘E’) appeared in the center of the computer screen for 1 s. The computer algorithm calculated visual acuity by using participant's answers to changing optotype size and orientation. Participants were asked to identify if the optotype E was oriented up, down, left or right. After the correct identification of at least three of five successive optotype orientations of a given size, the optotype size was reduced and the process was repeated until the orientation of the optotype could no longer be identified reliably**.** Thus, static visual acuity was based on the smallest optotype that could be identified accurately and reliably. This same algorithm was used for DVAT.

#### Perception timed test

The PTT measures the minimum amount of time in milliseconds that the optotype momentarily appearing on the computer screen is correctly and reliably identified by the participant. That is, the PTT measures the fastest time that a participant can reliably perceive the correct orientation of the optotype. The PTT establishes a participant-specific visual perceptual baseline to be used during the DVAT. To prevent a corrective saccade, participants were excluded if their PTT surpassed 80 ms [16,17]. This did not occur in the present study.

#### Dynamic visual acuity

The DVAT measures visual acuity that occurs with rhythmic head movement in pitch and yaw planes. The difference between DVAT and SVAT demonstrates the relative decrease in visual acuity that occurs as a result of head movement. For the DVAT, participants wore a headband that had an attached axis angular rate sensor (InterSense InertiaCube2, 3-axis, integrating gyroscope); this sensor was used to continuously monitor the velocity and position of head motion. In the pitch plane, participants moved their heads up and down in an arc of 40° (∼20° neck flexion and 20° neck extension) repeatedly as if indicating ‘yes’ at a velocity of between 160 and 220°/s. A metronome was used to assist the participant in maintaining the desired movement velocity (120 beats per min). All participants performed a practice trial of the DVAT procedure to familiarize themselves with the task and to minimize the learning effect.

Once the proper velocity and range of motion were maintained three-times consecutively, the optotype would appear for a short-time (derived from the PTT) and the participant reported the orientation of the randomly appearing optotype. A computer algorithm, based on identification of the correct optotype orientation (similar to the SVAT), generated the DVAT score. This same method was used for the yaw plane, except the participants rotated the head 20° to the left and 20° to the right as if indicating ‘no’.

### Data analysis

All statistical analyses were conducted using SPSS version 22.0 (IBM Corp., NY, USA) and using logMAR values; however, corresponding Snellen fraction values are also presented for additional perspective. Secondary to the small sample size, a Friedman's analysis of variance was used to compare the SVAT, DVAT in yaw and DVAT in pitch. If that analysis of variance was statistically significant, then two pairwise comparisons (SVAT to DVAT in yaw; and, SVAT to DVAT in pitch) would be conducted using Wilcoxon Signed Rank Tests and a Bonferroni-corrected α (α = 0.025). Pearson correlations were used to determine if movement velocity (deg/s) played a role in DVAT (logMAR values)**.** To compare the differential effects of concussion history on visual acuity decay with head movement, the difference scores (SVAT–DVAT) in both planes were calculated with the differences representing the decay in visual acuity with movement. Nonparametric Mann–Whitney U-tests were conducted to compare concussion history (yes and no) to the visual acuity drop. This was conducted for both yaw and pitch planes. All statistical tests, unless otherwise noted, were two-tailed with α = 0.05. There were no missing data for any of the participants.

## Results

### Overall

**Figure 1.**
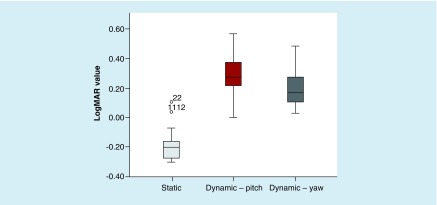
**Mean and 95% CIs of static and dynamic visual acuity in yaw and pitch.**

**Figure 2.**
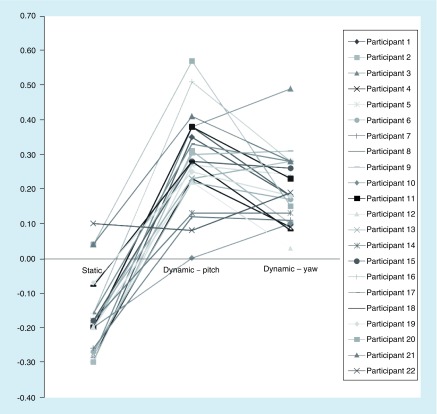
**Static and dynamic LogMAR values for each participant.**

There was a statistically significant difference among the SVAT, DVAT in yaw and DVAT in pitch, χ^2^ (2) = 44.000; p < 0.001 (Figure 1). The mean SVAT logMAR was -0.173 (SD = 0.114; 95% CI: -0.122 to -0.223; Snellen fraction = 20/13.4) with a range of -0.30 (Snellen fraction = 20/10.0) to 0.10 (Snellen fraction = 20/25.2). Data for individual participants can be found in Figure 2. Mean DVAT scores decreased with head movement to 0.196 logMAR (SD: 0.103; 95% CI: 0.151–0.242; Snellen fraction = 20/31.4; mean speed = 183.5°/s; SD: 13.5) in yaw and to 0.283 logMAR (SD: 0.133; 95% CI: 0.224–0.342; Snellen fraction = 20/38.4; mean speed = 177.8°/s; SD: 7.8) in pitch (Figure 1). Both the mean pitch and yaw DVAT were statistically significantly different from the SVAT mean (t(21) = -13.116; p < 0.001 and t(21) = -11.205; p < 0.001, respectively).

Movement velocities in both planes did not correlate with acuity loss (DVAT minus SVAT). Pitch speed did not correlate with the pitch acuity difference (r = 0.205; p = 0.360). Likewise, yaw speed did not correlate with the yaw acuity difference (r = 0.103; p = 0.648).

The pitch acuity difference (DVAT–SVAT = 0.455 ± 0.163) was greater than the yaw acuity difference (DVAT–SVAT = 0.369 ± 0.154), t(21) **=** -3.076, p = 0.006.

### Concussion history

**Figure 3.**
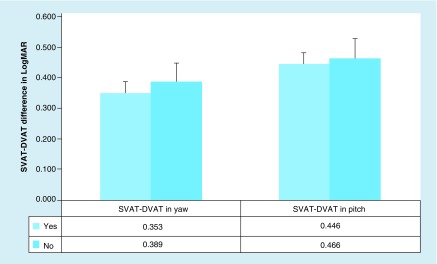
**Difference in visual acuity (mean and standard error of the mean) with head movement (static visual acuity test–dynamic visual acuity test) for those with (n = 12) and without (n = 10) a self-reported concussion history (yes or no) in pitch and yaw.** DVAT: Dynamic visual acuity test; SVAT: Static visual acuity testing.

There was not a statistically significant difference in the amount of visual acuity decay in those with (n = 12) and without (n = 10) a self-reported history of concussion in both the yaw (p = 0.691) and pitch (p = 0.448) planes (Figure 3).

## Discussion

Because this study was cross-sectional and there was not a control group, these results should be interpreted with caution and cause-and-effect relationships should not be inferred. Overall, the results of this study suggest that MMA fighters have significant decay in visual acuity with head movement in both the yaw and pitch planes. While, it is normal to have a loss of visual acuity with head movement, the loss of visual acuity observed in this study was more than what is commonly accepted by most healthcare practitioners as normal/subclinical (i.e., less than two levels on the Snellen fraction chart or logMAR level drop of less than 0.200). In the Li *et al*. normative DVAT data study, using a different but similar DVAT technique, these researchers found that the average decay in visual acuity for men in this same age profile was less than one Snellen fraction (age 18–29 mean logMAR = 0.084 ± 0.173; age 30–39 mean logMAR = 0.084 ± 0.169) [18]. Additionally, the decay in visual acuity in our study was considerably higher than was found in the Kaufman *et al*. study in college football players (0.248–0.254 logMAR) [14].

The losses of visual acuity observed in this study were greatest for pitch movements which went from -0.173 logMAR (Snellen fraction = 20/13) to 0.283 logMAR (Snellen fraction = 20/40). This represents a decrease of 0.456 logMAR. Yaw head movement caused a drop in visual acuity from -0.173 to 0.196 logMAR, which represents a decrease of 0.369 logMAR. Both of these decreases in visual acuity with head movement suggest VOR impairment. One logical reason for these larger than normal drops in visual acuity may be MMA-related cumulative head impact exposure; however, as was mentioned earlier, our study design was not appropriate for a cause-and-effect inference and we should, therefore, be cautious about this. From a practical relevance perspective, these results suggest that MMA participants in our study had a greater than normal loss of visual acuity with quick head movements as commonly happens in MMA participation; this loss of visual capacity could logically put them at greater risk for injury because quick head movements are common defensive maneuvers in MMA.

On the other hand, it is important to consider that dynamic visual acuity testing may not tell the whole story. First, dynamic visual acuity may be confounded by cognition and attention [19]; second, VOR suppression and alterations in VOR function have been reported across various populations, most notably in pilots. It is possible that this same phenomenon is occurring in MMA athletes wherein repeated vestibular stimulation (e.g., quick head movements) has triggered vestibular habituation. Last, while compensatory saccades can mask VOR impairment, anti-compensatory saccades could degrade dynamic visual acuity outcomes even in the presence of a normal functioning VOR [20]. Therefore, the conclusion that the visual acuity decrease observed in the present study is directly linked to VOR impairment may not be so straightforward. To gain a clearer picture, we recommend that researchers consider using video head impulse testing as it allows quantification of both a VOR deficit and the presence of head movement triggered saccades.

History of concussion did not appear to be related to SVA or DVA values which seems counterintuitive but was consistent with findings from another study in football athletes [14]. It would seem logical that those with a history of head trauma resulting in a concussion would have had more residual symptoms and, subsequently, a greater decay in visual acuity with head movement compared with those with no history of concussion. This has been shown in the acute [21] and chronic [22] stages after concussion but was not evident in the present study. It is possible that self-reported concussion history relative to subconcussive events is simply not a good indicator of the severity and extent of protracted brain injury. That is, both concussive and subconcussive head trauma may be causing similar long-term impairment. In fact, the present results may be suggestive of the cumulative dose effect of subconcussive events causing long-term residual deficits.

It should be noted that concussion history was self-reported in the present study and may have been under-reported (10 reported no concussion history and the mean number of concussions was 0.64). Systematic under-reporting of concussion symptoms has been reported in the literature [23]. Additionally, while athletes may have sustained a concussion they may have not reported it since it may not have been formally diagnosed by a healthcare practitioner. Meehan *et al*. reported that nearly a third of athletes have sustained previously undiagnosed concussions [24]. Also, Langlois *et al*. reported that the majority of concussions (81–92%) have more subtle signs and may go unrecognized as a concussion [25]. To further complicate matters, Abdullah *et al*. have discussed the complexity of concussion reporting in football which may share some similar stigma issues [26]. For instance, they suggest that a player reporting a concussion may be stigmatized for a lack of ‘toughness’. It is possible that this same stigma scenario may have happened in the reporting of concussion in the present study and, therefore, the true relationship between concussion history and long-term impact remains elusive.

One of the primary limitations of this study was the lack of a control group, small sample size and the narrowness of the sample which may limit its generalizability. Because of the lack of a control group, readers should be cautious about the interpretation of the results in the present study. However, one of the groups to which we were comparing our results (Kaufman *et al*.) [14] was conducted by the same research team and, therefore, used similar methods. While the Kaufman *et al*. study was a younger population, it was gender-matched and could be argued that the participant activity levels were similar. Also, the DVAT mean speeds were similar in both studies (177.4 and 176.1°/s in yaw and pitch in the Kaufman *et al*. study [14] and 183.5 and 177.8°/s in yaw and pitch in the present study). Another limitation is that participants were asked to report their concussion history in categorical terms (0 concussion [n = 10], 1–3 concussions [n = 11], 4–6 concussions [n = 0] and 6+ concussions [n = 1]). In retrospect, this lowered the level of measurement and limited our ability to run more probing analyses. Additionally, issues like concussion self-reporting may limit the confidence of interpreting their relationship to long-term residual symptoms. The date of the most recent concussion was not collected. It is possible that some residual symptoms may have been present; however, participants were excluded if they reported current symptoms from a concussion. Another limitation may be the potential for a learning effect. Li *et al*. 2014 suggested that they observed a significant learning effect with dynamic visual acuity testing even with a brief practice trial like we conducted in our study [18]. If a learning effect had indeed occurred in our study we would have observed better scores with tests administered later. As the pitch DVAT was always performed second in our study, we could assume that these DVAT values would benefit from the learning effect. However, the pitch scores were worse than the yaw scores which could mean that the pitch scores were actually worse without the learning effect.

## Conclusion

The results of this study suggest that actively training, asymptomatic MMA fighters have a significant decay in visual acuity with rapid head movements in both the yaw and pitch planes. While a loss of visual acuity with head movement is normal, the loss of visual acuity observed in this study was more than what is commonly accepted by most healthcare practitioners as normal. The decreases in visual acuity observed with head movement in this study suggest vestibulo-ocular reflex impairment. Additionally, there were no differences in the decay of visual acuity with movement for those with and without a history of concussion.

**Summary points**

- We investigated the amount of visual acuity loss during head movement in mixed martial arts (MMA) athletes.
- The difference between static visual acuity and dynamic visual acuity is one way to test the function of the vestibulo-ocular reflex (VOR).
- In total, 22 actively training and asymptomatic mixed martial arts athletes participated in this study.
- A significant decay, beyond what is considered normal ranges, in visual acuity was observed during dynamic head movement.
- The deficits observed may suggest VOR impairment which, in turn, may suggest that participation in MMA may expose these athletes to head trauma that negatively affects the complex interaction between the vestibular system and the visual system.
- Interestingly, there was no difference in dynamic visual acuity impairment between those with and without a self-reported concussion history.
- The lack of association between concussion history and dynamic visual acuity impairment may suggest that subconcussive head trauma in MMA is sufficient to affect the VOR; however, the complex nature of concussion self-reporting may obscure the true relationship.

## References

[1] Bledsoe GH, Hsu EB, Grabowski JG, Brill JD, Li G (2006). Incidence of injury in professional mixed martial arts competitions. *J. Sports Sci. Med.*.

[2] Scoggin JF, Brusovanik G, Pi M (2010). Assessment of injuries sustained in mixed martial arts competition. *Am. J. Orthop. (Belle Mead NJ)*.

[3] Lystad RP, Gregory K, Wilson J (2014). The epidemiology of injuries in mixed martial arts: a aystematic review and meta-analysis. *Orthop. J. Sports Med.*.

[4] Ngai KM, Levy F, Hsu EB (2008). Injury trends in sanctioned mixed martial arts competition: a 5-year review from 2002 to 2007. *Br. J. Sports Med.*.

[5] Zazryn TR, McCrory PR, Cameron PA (2008). Neurologic injuries in boxing and other combat sports. *Neurol. Clin.*.

[6] Buse GJ (2006). No holds barred sport fighting: a 10 year review of mixed martial arts competition. *Br. J. Sports Med.*.

[7] Corwin DJ, Wiebe DJ, Zonfrillo MR (2015). Vestibular deficits following youth concussion. *J. Pediatr.*.

[8] Guskiewicz KM (2011). Balance assessment in the management of sport-related concussion. *Clin. Sports Med.*.

[9] Marar M, McIlvain NM, Fields SK, Comstock RD (2012). Epidemiology of concussions among United States high school athletes in 20 sports. *Am. J. Sports Med.*.

[10] Guskiewicz KM, Broglio SP (2011). Sport-related concussion: on-field and sideline assessment. *Phys. Med. Rehabil. Clin. N. Am.*.

[11] Schubert MC, Minor LB (2004). Vestibulo-ocular physiology underlying vestibular hypofunction. *Phys. Ther.*.

[12] Gottshall K, Drake A, Gray N, McDonald E, Hoffer ME (2003). Objective vestibular tests as outcome measures in head injury patients. *Laryngoscope*.

[13] Ellis MJ, Leddy JJ, Willer B (2015). Physiological, vestibulo-ocular and cervicogenic post-concussion disorders: an evidence-based classification system with directions for treatment. *Brain Inj.*.

[14] Kaufman DR, Puckett MJ, Smith MJ, Wilson KS, Cheema R, Landers MR (2014). Test-retest reliability and responsiveness of gaze stability and dynamic visual acuity in high school and college football players. *Phys. Ther. Sport.*.

[15] Mohammad MT, Whitney SL, Marchetti GF, Sparto PJ, Ward BK, Furman JM (2011). The reliability and response stability of dynamic testing of the vestibulo-ocular reflex in patients with vestibular disease. *J. Vestib. Res.*.

[16] Leigh RJ, Brandt T (1993). A reevaluation of the vestibulo-ocular reflex: new ideas of its purpose, properties, neural substrate, and disorders. *Neurology*.

[17] Ward BK, Mohammad MT, Whitney SL, Marchetti GF, Furman JM (2010). The reliability, stability, and concurrent validity of a test of gaze stabilization. *J. Vestib. Res.*.

[18] Li C, Beaumont JL, Rine RM, Slotkin J, Schubert MC (2014). Normative scores for the NIH toolbox dynamic visual acuity test from 3 to 85 years. *Front. Neurol.*.

[19] Lee MY, Kim MS, Park BR (2004). Adaptation of the horizontal vestibuloocular reflex in pilots. *Laryngoscope*.

[20] Ramaioli C, Colagiorgio P, Saglam M (2014). The effect of vestibulo-ocular reflex deficits and covert saccades on dynamic vision in opioid-induced vestibular dysfunction. *PLoS ONE.*.

[21] Murray NG, Ambati VN, Contreras MM, Salvatore AP, Reed-Jones RJ (2014). Assessment of oculomotor control and balance post-concussion: a preliminary study for a novel approach to concussion management. *Brain Inj.*.

[22] Honaker JA, Criter RE, Patterson JN, Jones SM (2015). Gaze stabilization test asymmetry score as an indicator of previous concussion in a cohort of collegiate football players. *Clin. J. Sport Med.*.

[23] Meier TB, Brummel BJ, Singh R, Nerio CJ, Polanski DW, Bellgowan PS (2015). The underreporting of self-reported symptoms following sports-related concussion. *J. Sci. Med. Sport.*.

[24] Meehan WP, Mannix RC, O'Brien MJ, Collins MW (2013). The prevalence of undiagnosed concussions in athletes. *Clin. J. Sport Med.*.

[25] Langlois JA, Rutland-Brown W, Wald MM (2006). The epidemiology and impact of traumatic brain injury: a brief overview. *J. Head Trauma Rehabil.*.

[26] Abdullah KG, Grady MS, Levine JM (2015). Concussion and football: a review and editorial. *Curr. Neurol. Neurosci. Rep.*.

